# Astaxanthin and Docosahexaenoic Acid Reverse the Toxicity of the Maxi-K (BK) Channel Antagonist Mycotoxin Penitrem A

**DOI:** 10.3390/md14110208

**Published:** 2016-11-09

**Authors:** Amira A. Goda, Khayria M. Naguib, Magdy M. Mohamed, Hassan A. Amra, Somaia A. Nada, Abdel-Rahman B. Abdel-Ghaffar, Chris R. Gissendanner, Khalid A. El Sayed

**Affiliations:** 1 Department of Basic Pharmaceutical Sciences, School of Pharmacy, University of Louisiana at Monroe, 1800 Bienville Drive, Monroe, LA 71201, USA; amori65@yahoo.com (A.A.G.); gissendanner@ulm.edu (C.R.G.); 2 Department of Food Contaminant and Toxicology, National Research Center, Cairo 12622, Egypt; naguib_kh@hotmail.com (K.M.N.); hassanamra2010@yahoo.com (H.A.A.); somaianada@yahoo.com (S.A.N.); 3 Biochemistry Department, Faculty of Science, Ain Shams University, Cairo 11566, Egypt; mutation6119@yahoo.com (M.M.M.); abdo@abdoscience.com (A.-R.B.A.-G.)

**Keywords:** astaxanthin, BK (Maxi-K) channels, docosahexaenoic acid, food mycotoxin, penitrem A, toxicity

## Abstract

Penitrem A (PA) is a food mycotoxin produced by several terrestrial and few marine *Penicillium* species. PA is a potent tremorgen through selective antagonism of the calcium-dependent potassium BK (Maxi-K) channels. Discovery of natural products that can prevent the toxic effects of PA is important for food safety. Astaxanthin (AST) is a marine natural xanthophyll carotenoid with documented antioxidant activity. Unlike other common antioxidants, AST can cross blood brain barriers (BBBs), inducing neuroprotective effects. Docosahexaenoic acid (DHA) is polyunsaturated ω-3 fatty acid naturally occurring in fish and algae. DHA is essential for normal neurological and cellular development. This study evaluated the protective activity of AST and DHA against PA-induced toxicity, in vitro on Schwann cells CRL-2765 and in vivo in the worm *Caenorhbitidis elegans* and Sprague Dawley rat models. PA inhibited the viability of Schwann cells, with an IC_50_ of 22.6 μM. Dose-dependent treatments with 10–100 μM DHA significantly reversed the PA toxicity at its IC_50_ dose, and improved the survival of Schwann cells to 70.5%–98.8%. Similarly, dose-dependent treatments with 10–20 μM AST reversed the PA toxicity at its IC_50_ dose and raised these cells’ survival to 61.7%–70.5%. BK channel inhibition in the nematode *C. elegans* is associated with abnormal reversal locomotion. DHA and AST counteracted the in vivo PA BK channel antagonistic activity in the *C. elegans* model. Rats fed a PA-contaminated diet showed high levels of glutamate (GLU), aspartate (ASP), and gamma amino butyric acid (GABA), with observed necrosis or absence of Purkinjie neurons, typical of PA-induced neurotoxicity. Dopamine (DA), serotonin (5-HT), and norepinephrine (NE) levels were abnormal, Nitric Oxide (NO) and Malondialdehyde (MDA) levels were significantly increased, and total antioxidant capacity (TAC) level in serum and brain homogenates was significantly decreased in PA-treated rats. DHA and AST treatments effectively counteracted the toxic effects of PA and normalized most biochemical parameters in rats. DHA and AST can be useful food additives to prevent and reverse PA food-induced toxicity.

## 1. Introduction

Penitrem A (PA) is a halogenated indole alkaloidal mycotoxin produced by the fungus *Penicillium crustosum* and several other terrestrial and few marine *Penicillium* species [1]. PA is one of a group of tremor-producing agents through its selective antagonistic effect on the calcium-dependent Maxi-K (BK) potassium channels in brain [2]. The most toxic fungus *P*. *crustosum* occurs frequently in spoiled food and feed and produces a large number of secondary metabolites and mycotoxins; in addition to PA, several other penitrem analogues and precursors are produced [3]. PA is the most potent tremorgen and BK channel antagonist among the penitrems. *P. crustosum* is found in nuts [4], fruits, meats, cereals [5], and cheeses [6]. The growth substrate had a distinctive effect on the mycotoxin production ability of this fungus. For example, *P. crustosum* produces considerable amounts of PA when fermented on cheese due to the high protein and amino acid contents of this substrate required for the biosynthesis of PA [7]. Several PA-induced neurological effects have been reported in animals during the past 30 years [8]. A few cases of human neurotoxicities have also been linked with penitrems [9]. Even though the primary target of PA is the central nervous system (CNS), the exact mechanism of PA’s action in brain is still unknown [10]. Neuropharmacological studies of PA showed its selective excitatory and inhibitory effects on neurotransmitters in the CNS [10].

Astaxanthin (AST) is a natural marine xanthophyll keto-carotenoid occurring in microalgae, plankton, krill, and seafood. AST is responsible for the distinctive reddish coloration of salmon, trout, and crustaceans such as shrimp and lobster. It also occurs in several yeasts, fungi, plants, and the feathers of some birds, including flamingos and quail [11]. AST attenuates oxidative stress and inflammation mediators, and showed beneficial effects in non-cardiovascular disease [12]. In addition, AST decreased the markers of lipid peroxidation [13], inflammation [14], and thrombosis [15]. AST exerts multiple protective effects in the brain, because it is capable of crossing blood–brain barriers (BBBs), unlike many other antioxidant molecules [16].

Docosahexaenoic acid (DHA, all-*cis*-docosa-4,7,10,13,16,19-hexa-enoic acid) is a 22-carbon ω-3 fatty acid commonly found in cold-water fish oil , and is commercially produced by the microalgae *Crypthecodinium cohnii* and *Schizochytrium* species [17]. DHA is a major component of brain gray matter and of the retina in most mammalian species, and is considered essential for normal neurological and cellular development [18]. There has been recent interest in DHA, particularly concerning its promising role as a micronutrient for neurodevelopment, neuro-recognition, and neurodegeneration disorders [19]. DHA also showed an emerging preventive role for neuropsychiatric disorders such as psychosis [19] and affective disorders [20].

The aim of this study is to reverse or prevent toxicities expected to result from the exposure to PA contaminants in foods by using two common marine natural products, AST and DHA. Therefore, this study evaluated the protective activity of AST and DHA against the in vitro PA-induced toxicity using Schwann cells CRL-2765 (in vitro model), and the nematode *C. elegans* and Sprague Dawley rats in vivo models.

## 2. Results

### 2.1. Schwann Cells (RSC 96 ATCC CRL-2765) In Vitro Model

#### 2.1.1. In Vitro Effects of PA on Neuronal Schwann Cell (RSC 96 ATCC CRL-2765) Proliferation and Survival

**Figure 1:**
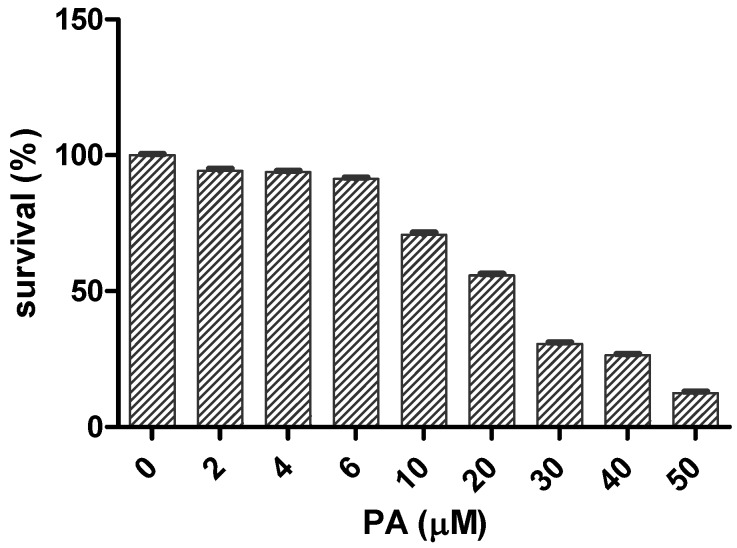
Dose-dependent survival of Schwann cells upon exposure to increasing concentrations of penitrem A (PA) for 24 h. All values are relative to the vehicle control (100% survival). Values presented as mean (*n* = 4) in triplicate *p* < 0.05, log IC_50_ = 1.354.

Exposure of Schwann cells RSC 96 [21] to increasing concentrations of PA for 24 h resulted in significant dose-dependent suppressive effects on these cells’ survival, with an IC_50_ of 22.6 μM (Figure 1). PA at a concentration of 4 μM and higher induced a statistically significant decrease in survival (*p* < 0.05) of the Schwann cells in comparison to the vehicle-treated control. At a concentration of 50 μM PA, only 12.6 ± 0.5% (mean ± SEM, *p <* 0.05) of the cells survived, in comparison to the vehicle-treated control.

#### 2.1.2. Protective Effects of AST and DHA against the PA-Induced Toxicity to Schwann Cells

**Figure 2:**
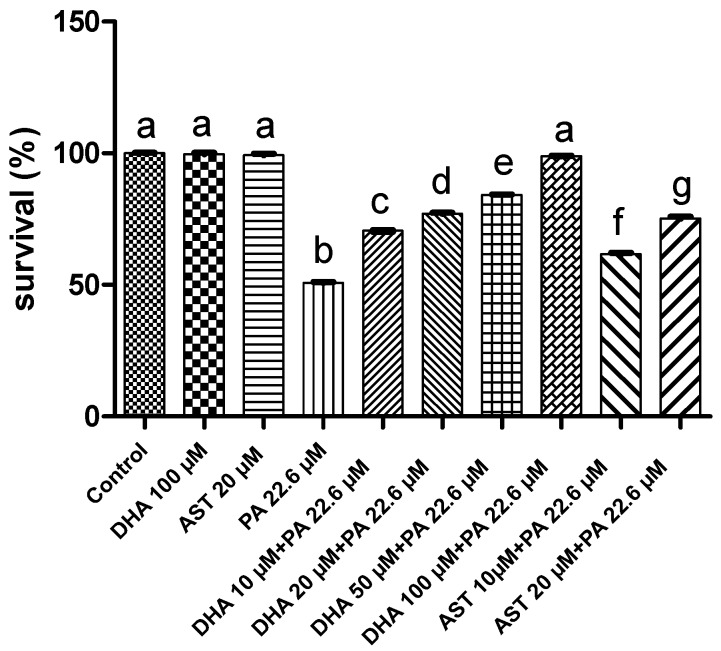
Protective effects of combined DHA and AST treatments against the cytotoxicity of PA at its IC_50_ value (22.6 μM) on Schwann cells. Treatment groups compared with the vehicle-treated control (DMSO) groups. Error bars indicate SEM for at least *n* = 4 in triplicate, *p* < 0.05, compared to their respective DMSO-treated control group. Different letters (a–g) indicate significant differences (*p* ≤ 0.05). PA = penitrem A, AST = astaxanthin, DHA = docosahexaenoic acid; DMSO: dimethyl sulfoxide.

PA at a dose of 22.6 μM decreased the Schwann cells’ survival to 49.8%, compared to the vehicle control dimethyl sulfoxide (DMSO) group. Two different treatment doses of DHA and AST were used to study potential protective effects against the PA-induced cytotoxicity to Schwann cells. Combined 10–100 μM DHA treatments with 22.6 μM PA raised the percent Schwann cell survival to 70.5%–98.8%, respectively (Figure 2). Similarly, combined 10 μM or 20 μM treatments of AST with 22.6 μM PA treatment raised the percent Schwann cell survival to 61.7% and 75.2%, respectively.

### 2.2. Caenorhbditis elegans In Vivo Model

The nematode *C. elegans* is a valid in vivo model with numerous advantages, including the availability of the complete *C. elegans* genome sequence, rapid growth rate, ease of culturing, short life cycle, and the correlation of many basic physiological processes and stress responses with higher organisms (e.g., humans). This makes *C. elegans* a genetically tractable model to dissect cellular processes in vivo. BK channel inhibition in the nematode *C. elegans* is associated with abnormal locomotion behavior. This behavior includes an increase in the number of times the worm stops and reverses its direction, which can be easily assessed and quantified. This model was used to assess the PA-induced BK channel antagonistic effects with a broad dose range (1–50 μM) and known documented activity [22,23,24,25].

#### DHA and AST Counteracted PA’s BK Channel Antagonistic Activity in *C. elegans* Model

**Figure 3:**
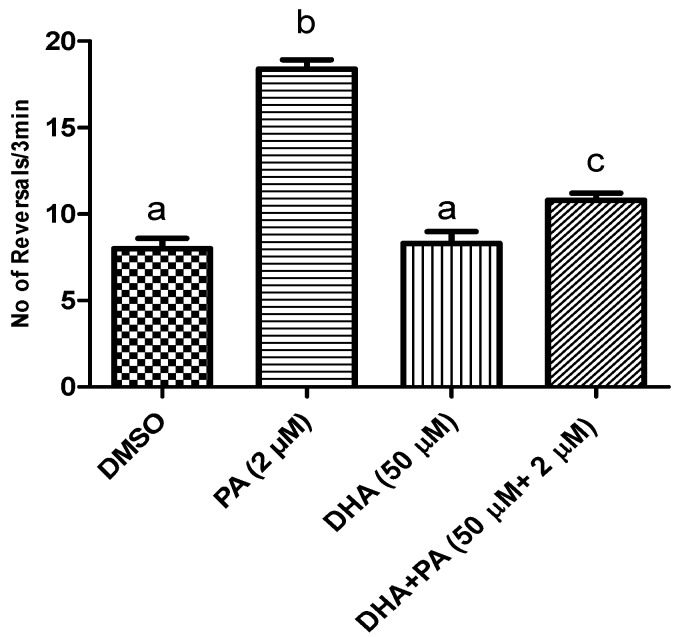
The protective activity of DHA against PA’s BK (Maxi-K) channel inhibitory effects in *C. elegans* model. Wild type N2 strain treated with 50 μM DHA, 2 μM PA, and combined DHA (50 μM) and PA (2 μM) treatments. Treatment groups were compared with DMSO (vehicle control). Error bars indicate SEM for at least *n* = 12. *p* < 0.05 compared to their respective DMSO-treated control group. Different letters (a–c) indicate significant differences (*p* ≤ 0.05). PA = penitrem A, AST = astaxanthin, DHA = docosahexaenoic acid.

**Figure 4:**
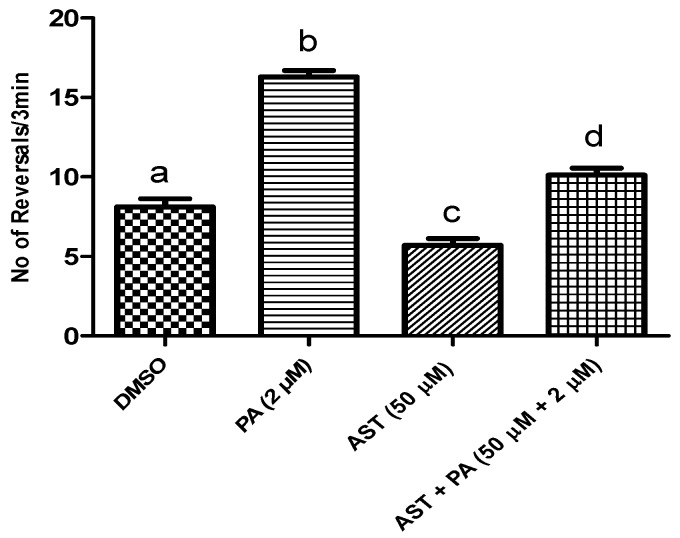
The protective activity of astaxanthin (AST) against the PA’s BK channel inhibitory effects in *C. elegans* model. Wild type N2 strain treated with AST 50 μM, PA 2 μM, and combined AST 50 μM with PA 2 μM. Error bars indicate SEM for at least *n* = 12. *p* < 0.05 compared to their respective DMSO-treated control group. Different letters (a–d) indicate significant differences (*p* ≤ 0.05). PA = penitrem A, AST = astaxanthin, DHA = docosahexaenoic acid.

We used this experimental system to assess the ability of DHA and AST to protect the worm against PA’s BK channel antagonistic effect. A PA dose of 2 μM was selected based on previous studies [22,23,24]. Combined treatment of 50 μM DHA with 2 μM PA significantly reduced the number of *C. elegans* reversals, suggesting the counteraction of PA’s BK channel antagonistic effects (Figure 3). Similarly, 50 μM AST treatment reduced the number of *C. elegans* reversals, and hence counteracted the PA BK channel antagonistic activity (Figure 4).

### 2.3. Sprague Dawley Rat In Vivo Model

Three-month old Sprague Dawley rats (100–120 g) were purchased and acclimated to animal house facility conditions at a temperature of 18–25 °C, with a relative humidity of 55% to 65%, and a 12-h light/dark cycle for one week prior to the experiments. The animals were maintained on a standard laboratory diet (protein: 160.4, fat: 36.3, and fiber 41 g/kg) and had free access to food and water. Animals were divided into eight groups (10 rats/group), housed in filter-top polycarbonate cages, and maintained on their respective diet for four weeks. The maximum oral dose of PA for severe spontaneous tremor was 8 mg/kg body weight [26]. PA-treated groups were fed on high PA diet containing 46.5 mg/kg diet. AST-treatment group animals were administered AST dissolved in corn oil by oral gavage at a dose of 12 mg/kg body weight. Similarly, DHA-treated group animals were administered the treatment in corn oil at a 250 mg/kg body weight dose using oral gavage. The animals were observed daily for signs of toxicity and visually observed tremorgenic syndromes after four weeks. Animals were euthanized by cervical dislocation after blood sample collection, and brain samples were dissected and homogenized for the determination of neurotransmitters, total antioxidant capacity (TAC), malondialdehyde (MDA), and nitric oxide (NO) levels. Another portion of each animal brain was dissected and fixed in 10% formalin for histopathological studies.

#### 2.3.1. DHA and AST Counteract the Toxic Effects of PA on Amino Acid and Monoamine Neurotransmitters in Sprague Dawley Rats

**Figure 5:**
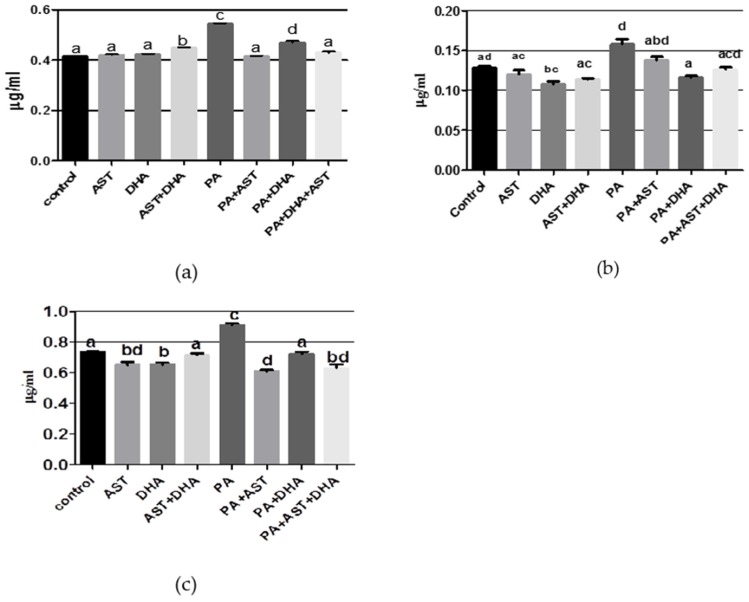
Effects of monotherapy and combined treatments of DHA and AST on PA-induced amino acid and neurotransmitter levels in Sprague Dawley rat brain homogenates. (**a**) aspartate; (**b**) glutamate; (**c**) GABA levels. Different letters (a–d) indicate significant differences (*p* ≤ 0.05). PA = penitrem A, AST = astaxanthin, DHA = docosahexaenoic acid.

**Figure 6:**
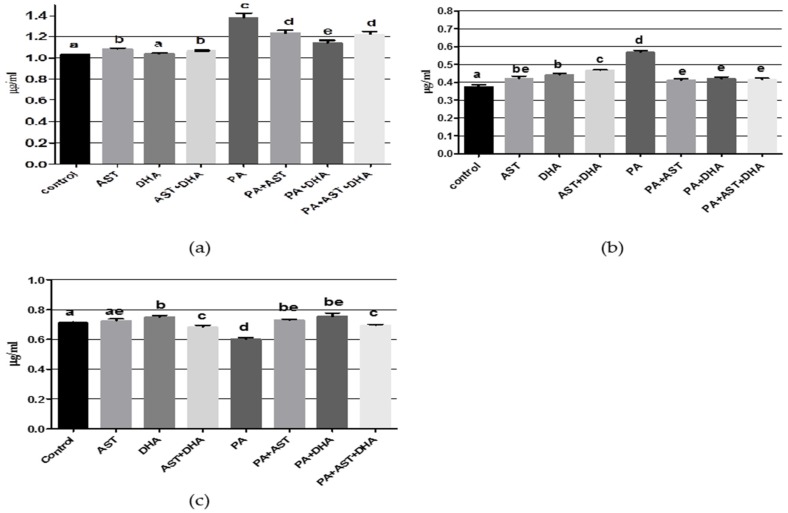
Effects of monotherapy and combined treatments of DHA and AST on PA-induced monoamines neurotransmitter levels in Sprague Dawley rat brain homogenates. (**a**) dopamine; (**b**) serotonin (5-HT); (**c**) norepinephrine. Different letters (a–e) indicate significant differences (*p* ≤ 0.05). PA = penitrem A, AST = astaxanthin, DHA = docosahexaenoic acid.

The AST monotherapy control group had normal values of aspartate, glutamate, and norepinephrine (NE); however, there was significant inhibition in gamma amino butyric acid (GABA) and elevation of the dopamine (DA) and 5-hydroxytryptophan (5-HT) values, when compared with vehicle control group (Figure 5 and Figure 6). The DHA monotherapy-treated group had significantly lower glutamate and GABA levels, and significantly elevated NE and 5-HT in brain homogenate, when comparing with the vehicle control values (Figure 5 and Figure 6). Combined treatment of AST + DHT exhibited significant elevation in aspartate, DA, and 5-HT levels, in addition to significantly reduced glutamate and NE levels in brain homogenates, in comparison with the vehicle control group (Figure 5 and Figure 6). The PA-fed group showed significant elevation in aspartate, glutamate, GABA, DA, and 5-HT levels, while only NE significantly decreased in brain homogenate, compared to the other treatment and vehicle control groups. Combined treatment of PA with AST normalized aspartate level (Figure 5a) and significantly decreased GABA level (Figure 5c), and increased NE level (Figure 6c). AST/PA treatment showed significant improvement in glutamate (Figure 5b), dopamine (Figure 6a), and 5-HT levels (Figure 6b) when compared with the PA-treated group. Moreover, combined DHA/PA treatment significantly reduced glutamate level (Figure 5b), elevated NE levels (Figure 6c), and normalized GABA (Figure 5c), DA (Figure 6a), and 5-HT (Figure 6b) levels, compared with PA-treated group. AST + DHA/PA treatment normalized aspartate and glutamate levels, significantly decreased GABA and NE levels, and ameliorated the PA-induced elevated DA and 5-HT levels, when compared with PA-fed group.

#### 2.3.2. DHA and AST Significantly Increased TAC Value in PA-Treated Sprague Dawley Rat Serum and Tissue Homogenates

**Figure 7:**
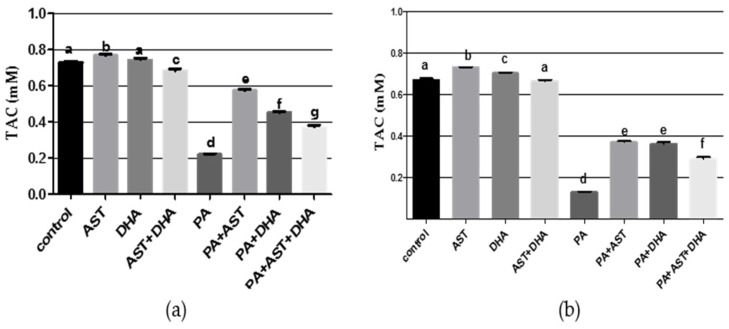
Effects of monotherapy and combined AST and DHA on total antioxidant capacity (TAC) individually and combined with PA in Sprague Dawley rat serum or tissue homogenates. (**a**) Tissue TAC; (**b**) Serum TAC. Different letters (a–g) indicate significant differences (*p* ≤ 0.05). PA = penitrem A, AST = astaxanthin, DHA = docosahexaenoic acid.

PA is known to induce oxidative damage and reduce total antioxidant capacity (TAC) level. TAC was significantly reduced in Sprague Dawley rat serum or tissue homogenates in the PA-fed group (Figure 7). AST and DHA monotherapy-treated groups had significantly elevated TAC in the animal’s serum or tissue homogenates, but TAC was slightly reduced in combined AST and DHA-treated group (Figure 7). Monotherapy and combined AST and DHA treatments significantly increased TAC values in serum and tissue homogenates of PA-treated animals compared to the group treated with only PA (Figure 7).

#### 2.3.3. DHA and AST Significantly Decreased the Oxidative Stress Markers Nitric Oxide (NO) and Malondialdehyde (MDA) in PA-Treated Sprague Dawley Rat Serum or Tissue Homogenates

**Figure 8:**
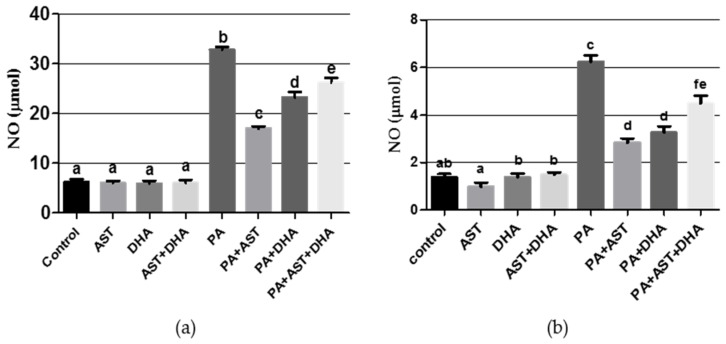
Effects of monotherapy and combined AST and DHA on nitric oxide (NO) levels individually and combined in PA-treated Sprague Dawley rat brain homogenates and serum. (**a**) brain homogenates NO; (**b**) serum NO. Different letters (a–f) indicate significant differences (*p* ≤ 0.05). PA = penitrem A, AST = astaxanthin, DHA = docosahexaenoic acid.

**Figure 9:**
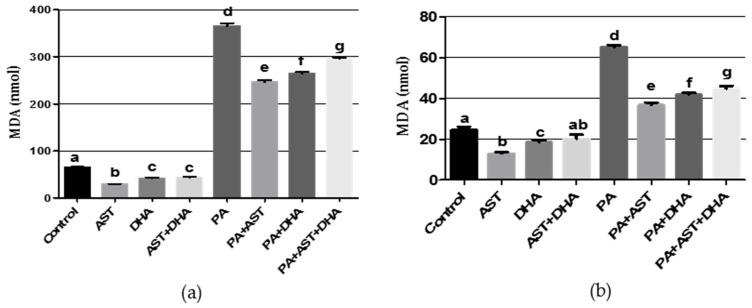
Effects of monotherapy and combined AST and DHA on malondialdehyde (MDA) levels individually and combined in PA-treated Sprague Dawely rat brain homogenates and serum. (**a**) brain homogenates MDA; (**b**) serum MDA. Different letters (a–g) indicate significant differences (*p* ≤ 0.05). PA = penitrem A, AST = astaxanthin, DHA = docosahexaenoic acid.

PA is known to induce oxidative stress, and is therefore expected to increase the levels of oxidative stress markers NO and MDA in animal models. PA treatment significantly increased NO and MDA levels in Sprague Dawely rat serum or brain homogenates (Figure 8 and Figure 9). Single or combined treatment with AST and/or DHA did not affect the base levels of NO and MDA in Sprague Dawley rats, compared to vehicle-treated control (Figure 8 and Figure 9). Monotherapy and combined AST and DHA-treatments significantly decreased NO and MDA levels in serum and brain homogenates of PA-treated Sprague Dawley rat, compared to individual PA-treated group (Figure 8 and Figure 9).

#### 2.3.4. AST and DHA Prevented PA-Induced Histopathological Changes in Sprague Dawely Rat Brain (Cerebrum and Cerebellum)

**Figure 10:**
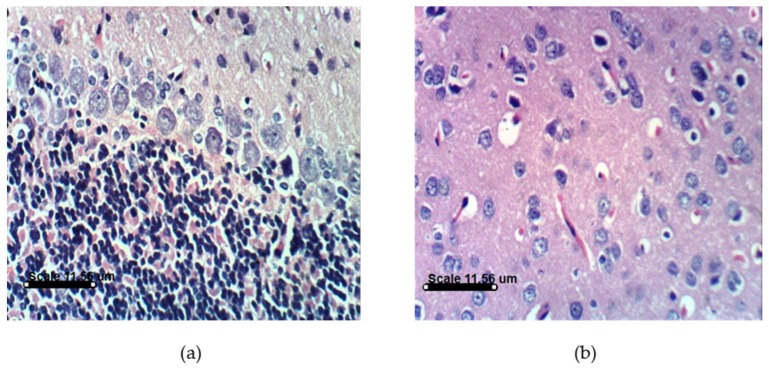
Histology of brain section for vehicle-treated control group rats: (**a**) Cerebellum section showing no histopathological changes with normal Purkinje cells pattern (H & EX400); (**b**) Cerebrum section of vehicle-treated control rats showing normal histological pattern (H & EX400).

**Figure 11:**
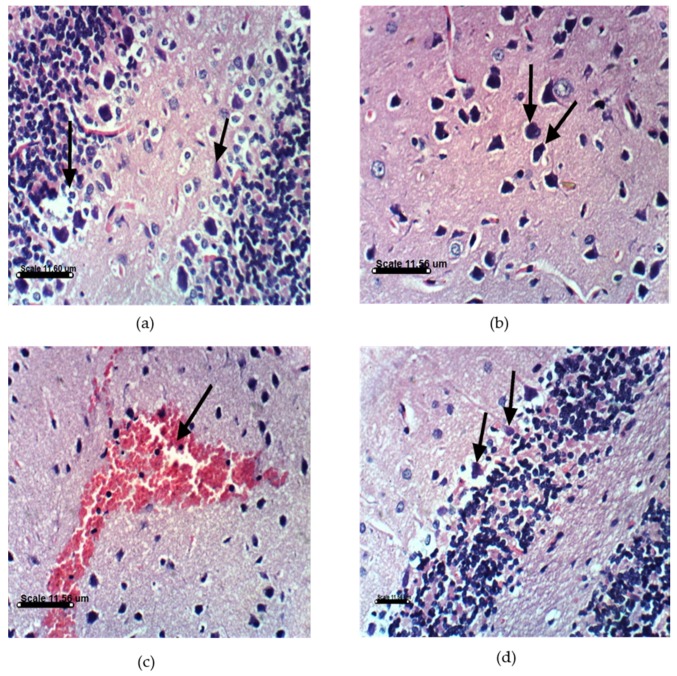
Histopathology of PA-treated rat brain sections. (**a**) PA-treated rat cerebellum section showing necrosis of Purkinje cells and vacuolization of molecular cell layer (H & EX400); (**b**) PA-treated rat cerebrum section showing necrosis of neurons and neuronophagia (H & EX400); (**c**) PA-treated rat cerebrum section showing focal cerebral hemorrhage (H & EX400); (**d**) PA-treated rat cerebellum section showing necrosis or even absence of Purkinje cells (H & EX100). PA = penitrem A.

**Figure 12:**
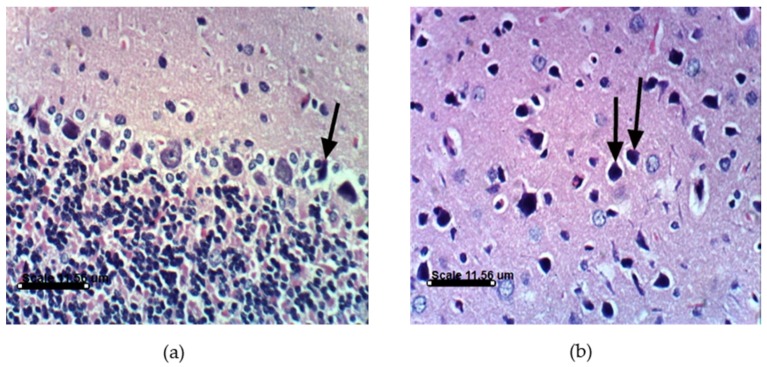
Histopathology of AST and PA-treated rat brain sections. (**a**) AST and PA-treated rat cerebellum section showing necrosis of some Purkinje cells, whereas other cells are normal (H & EX400); (**b**) AST and PA-treated rat cerebrum section showing necrosis of some neurons (H & EX400). PA = penitrem A, AST = astaxanthin.

**Figure 13:**
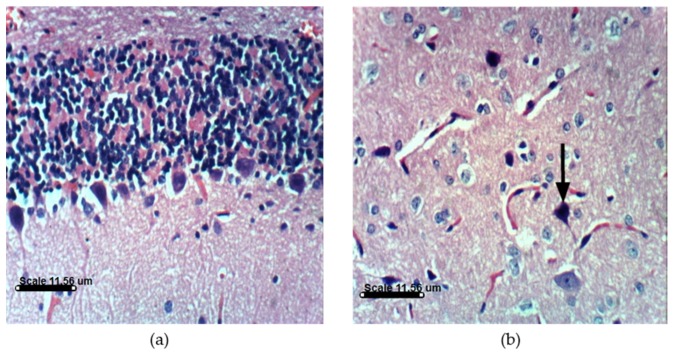
Histopathology of DHA and PA-treated rat brain sections. (**a**) DHA and PA-treated rat cerebellum section showing normal Purkinje cells (H & EX400); (**b**) DHA and PA-treated rat cerebrum section showing necrosis of some neurons (H & EX400). PA = penitrem A, DHA = docosahexaenoic acid.

**Figure 14:**
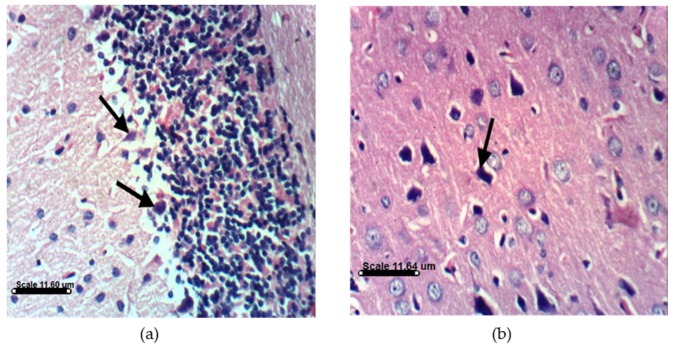
Histopathology of combined DHA, AST, and PA-treated rat brain sections. (**a**) DHA, AST, and PA-treated rat cerebellum section showing necrosis with decreased number of Purkinje cells (H & EX400); (**b**) DHA, AST, and PA-treated rat cerebrum section showing necrosis of neurons (H & EX400). PA = penitrem A, AST = astaxanthin, DHA = docosahexaenoic acid.

PA treatments in animals induce distinct histopathological and histochemical changes. Histological examination of the vehicle-treated Sprague Dawely rats control brain cerebellum and cerebrum showed normal histological and Purkinjie cells patterns (Figure 10a,b and Figure S1). Monotherapy and combined AST and DHA and vehicle-treated control rats also showed no histopathological changes (Figures S2–S4). Histopathological examination of the rat brains fed a PA-contaminated diet showed necrosis of Purkinje cells, vacuolization of molecular cell layer, and necrosis or even absence of Purkinje cells in cerebellum section (Figure 11a,d). They also showed necrosis of neurons and neurophagia in cerebrum sections (Figure 11b and Figure S5) and focal cerebral hemorrhage in cerebrum sections (Figure 11c). Monotherapy and combined AST and DHA-treatments markedly improved and nearly normalized the pathological changes of the PA-treated rat cerebellum and cerebrum sections (Figure 12, Figure 13 and Figure 14 and Figures S6–S8).

## 3. Discussion

The main peripheral nervous system glia is Schwann cells, which play a major role in the formation and regeneration of axons. Hence, Schwann cells represent an excellent in vitro cell culture model to study neurotoxicity [21]. In addition to its BK channel antagonistic effects, PA inhibited the proliferation of the human triple negative breast cancer MDA-MB-231 cells via the suppression of the total cellular β-catenin levels [22]. The purpose of this study is to assess the ability of DHA and AST to prevent and reverse PA toxicity in different models. PA dose-dependently inhibited the viability and proliferation of Schwann cells, with an IC_50_ of 22.6 μM (Figure 1). Combined treatment of Schwann cells with PA at its IC_50_ dose with a range of DHA treatments (10–100 μM) significantly prevented the PA toxicity, and improved the survival of Schwann cells to 70.5%–98.8% (Figure 2). Similarly, combined 10–20 μM treatments of AST with PA at its IC_50_ dose prevented its toxicity and raised these cells’ survival to 61.7%–70.5%. The nematode *C. elegans* was used to assess the PA BK channel inhibition and protective activities of DHA and AST [22,23,24,25]. BK channel inhibition in *C. elegans* is associated with abnormal worm locomotion behavior: increased reversal locomotion (i.e., increased the number of times in which the worm stops and reverses its direction). This number can be easily recorded and quantified to reflect the degree of BK channel antagonism [22,23,24]. PA showed BK channel inhibitory activity in the *C. elegans* model in treatment levels as low as 1 μM, based on the observed increased reversals number exhibited by treated worms compared to the vehicle (DMSO) control [22,23]. The long chain polyunsaturated ω-3 fatty acid DHA was recently reported as a BK channel agonist, reversibly activating the human large conductance Ca^2+^- and voltage gated K^+^ (Slo-1 BK) in cell free patches at 500 nM concentration, and hence proposed as a hypotensive entity [25]. DHA enhanced the BK channel activity in a β-subunit-dependent manner [27]. Therefore, DHA was able to lower the blood pressure of the wild type, but not the slo-1 knockout mice [28]. In the current study, DHA treatment counteracted PA’s BK channel antagonistic effects in the *C. elegans* model by showing a significant decrease in the number of the PA/DHA treated worm reversals, compared with PA-treated worm (Figure 3).

Similarly, although not reported as a direct BK channel agonist like DHA, AST has been reported to decrease blood pressure values, improving cardiovascular remodeling and oxidative stress [28]. BK channel activity influences blood pressure by setting vascular tone [29]. In the current study, 50 μM treatment of AST significantly decreased the number of reversals in *C. elegans* treated with 2 μM PA, compared to individual PA-treated worms (Figure 4). Therefore, AST is proposed to provide protection and was able to reverse PA-induced BK channel blocking toxicity in the *C. elegans* model.

The results of the rodent study indicated that PA-treated animals showed a significant increase in the levels of aspartate (Asp), glutamate (Glu), GABA, DA, and 5-HT, along with a significant decrease in the NE level (Figure 4 and Figure 5). The voltage and Ca^2+^-activated K^+^ channels are involved in the regulation of neurotransmitter release and neuronal excitability [30]. The PA-induced BK channel antagonistic effect may correlate with and justify the abnormality in Asp, Glu, GABA, DA, 5-HT, and NE levels in the PA-treated group. The higher dopamine level in the PA-treated group compared to vehicle-treated group might be a self-defense mechanism to protect against the tremor via the activation of dopaminergic neurons [31]. The higher DA level might be explained by a possible adrenergic link involved in the PA-induced tremor [31].

A growing body of evidence collected from epidemiological, clinical, and pre-clinical studies suggests that nutraceuticals, including the ω-3 polyunsaturated fatty acid DHA, have a protective effect on the development of some features associated with neurodegenerative diseases [32]. Over the last decade, several groups have reported the neuroprotective action of DHA against dopaminergic neuronal degeneration in animal models [33]. Multiple reports showed DHA’s mechanisms of action via its impact on neuronal functions, leading to neuro-rescue, neuro-restoration, and functional recovery [33,34,35]. DHA exhibited anti-inflammatory and anti-oxidant properties, also acting as a stimulator of the cellular signaling pathways involved in neuronal survival [36]. DHA supplementation enhanced the recovery of the DA-ergic system, restored the monoamine neurotransmission, and normalized hypothalamic–pituitary–adrenal axis (HPA) function in MDA [37]. DHA supplementation also enhanced serotoninergic and dopaminergic neurotransmission, decreased the levels of several HPA axis hormones in mice, and showed antidepressant-like effects in animal models [37]. Combined DHA treatment with PA in this study significantly inhibited the glutamate level, elevated NE (Figure 6c) level, and normalized GABA (Figure 5c), DA (Figure 6a), and 5-HT (Figure 6b) levels, compared to the PA-treated group.

AST improved the oxidative stress markers in most of the brain region by improving the antioxidant enzymes in various disease models [38]. AST maintained the viability of dopaminergic neuronal SH-SY5Y cells as assessed by the MTT assay [39], which may justify the higher DA level in the AST-treated group versus the vehicle control group. Combined treatment of AST with PA normalized the aspartate level, significantly decreased the GABA level (Figure 5c), and increased the NE level (Figure 6c). Combined AST with PA treatment also showed significant improvement in glutamate (Figure 5b), DA (Figure 6a), and 5-HT (Figure 6b) levels versus the PA-treated group. Combined AST and DHA with PA treatments effectively counteracted the effect of PA on the aspartate, glutamate, and GABA levels. They also restored the normal DA and 5-HT levels. The counteracting effect of AST and DHA for PA-induced BK channel antagonistic activity in the *C. elegans* model might have restored the normal levels of targeted neurotransmitters. Oxidative stress induces BBB permeability, promoting oxidative damage, mediating mitochondrial injury, and exacerbating neuro-inflammation. AST has been shown to increase the level of antioxidant enzymes in various disease models [39]. The decrease in the TAC in the PA-treated rats might indirectly increase the oxidative damage of DNA. Monotherapy and combined DHA and AST treatments with PA significantly raised the TAC values (Figure 7) compared to the PA-treated group. NO generation by inducible nitric oxide synthase (iNOS) plays a key role in cytokine-mediated cell destruction. DHA suppressed the expression of the iNOS gene and the production of NO [40]. Combined treatment of DHA or AST with PA reduced PA-induced NO generation (Figure 8). Individual use of DHA or AST with PA was more effective in preventing NO generation than the combined use of both compounds with PA treatment.

Lipid peroxidation damages biological membranes, leading to changes in membrane fluidity and function. Many lipid peroxidation products exert cytotoxic effects and alter cell signaling. AST’s chemical structure has two oxygenated groups on each ring structure, which enhances its antioxidant capacity. AST is a potent free radical scavenger, potent quencher of reactive oxygen, chain breaking antioxidant, including singlet oxygen and one-and two-electron oxidants [41]. In the current study, PA administration enhanced lipid peroxidation, as indicated by the significant increase in MDA level (Figure 9), which directly correlates with free radical-mediated toxicity. Combined treatment of AST or DHA with PA resulted in a significant improvement in MDA level.

The other important findings of the present study are notable improvements in the cerebellum and cerebrum sections in PA-treated groups when combined with either AST or DHA treatments. An important signature of neurological disorders is the significant loss of Purkinjie cells [42]. PA selectively damages the cerebellum via exocitotoxic actions on the Purkinjie cells in the vermis, producing a syndrome characterized by sustained tremors [8]. The GABA-ergic Purkinjie cells are especially sensitive to PA’s actions, where signs of ischemic neuronal death have been observed [42]. The results of this study are in agreement with literature animal data that showed partial loss of Purkinjie cells in PA-treated rats. PA-treated rat cerebellum sections in this study showed necrosis of the Purkinje cells and vacuolization of molecular cell layer (Figure 11a). Some sections occasionally showed an absence of Purkinjie cells (Figure 11d). Cerebrum sections of the PA-treated group showed necrosis of neurons, neuronophagia, and focal cerebral hemorrhage, compared to the vehicle-treated control group. AST reduced brain cell death [43]. DHA was previously reported to protect against aluminum-induced alteration of rat brain Purkinjie cells and granular layer [44]. AST and DHA succeeded in counteracting the PA-induced oxidative stress markers, which may justify the improvements in brain sections of PA-treated group by co-treatments with AST (Figure 12) or DHA (Figure 13). It is also evident that brain sections of either AST with PA or DHA with PA showed better improvement profiles compared to the triple combination treatments of PA, DHA, and AST (Figure 14), suggesting the preference to use either AST or DHA for in vivo prevention of PA-induced brain toxicity.

## 4. Materials and Methods

### 4.1. Chemicals

Standard PA was purchased from Sigma-Aldrich Chemical Co. (St. Louis, MO, USA). AST was purchased from Nutrex Hawaii Inc. Kailua-Kona, HI, USA. DHA was purchased from DSM Nutritional Products, Columbia, MD, USA.

### 4.2. PA Production

PA (95% HPLC purity) was produced by three-week static fermentation of iTEM 7046 *Penicillium crustosum* on SPS media (skimmed milk 2%–potato extract 2%–sucrose 2%) or on sterilized barley in 5 L Erlenmeyer flasks at 25 °C using the method described by El Banna and Leister (1988) [5].

### 4.3. PA Isolation and HPLC Quantitation

PA was extracted following the method described by Rundberget and Wilkins 2002b [45]. About 20 g fermented barley flasks were grinded and extracted with CH_3_CN–H_2_O (9:1, *v*/*v*) and homogenized for 1 h using an automatic shaker. The homogenates were filtered, and the crude extracts were evaporated under vacuum to dryness. The residue was reconstituted in 5 mL CH_3_CN–H_2_O (7:3, *v*/*v*) and subjected to preparative C-18 column (250 mm × 10 mm, 5 μm) reversed phase HPLC purchased from a Phenomenex, Torrance, CA, USA. PA was eluted at 4 mL/min using gradient elution starting with CH_3_CN–0.05 mM CH_3_COONH_4_ (4:6, *v*/*v*), which increased over 20 min to 9:1 (*v*/*v*) at λ_max_ 223 and 295 nm. The retention time of PA was 1.2 min.

### 4.4. Proliferation Assay

Schwann cells CRL 2765 (ATCC, Manassas, VA, USA) were plated at a density of 1 × 10^4^ cells per well (6 wells/group) in 96-well culture plates using the ATCC-recommended media (Dulbecco’s Modified Eagle’s Medium, DMEM), and allowed to adhere overnight for growth studies [23]. Next day, cells were divided into different groups and fed serum-free DMEM media supplemented with experimental treatments or vehicle-treated control media. Viable cell count was determined using the MTT colorimetric assay. The absorbance was measured at 570 nm on a BioTek Synergy2 microplate reader (BioTek, Winooski, VT, USA). The number of cells per well was calculated against a standard curve prepared at the start of the experiment. The IC_50_ value for each tested sample was calculated by nonlinear regression of log concentration versus the % survival, implemented in GraphPad PRISM version 5.0, GraphPad Software, Inc, La Jolla, CA, USA.

### 4.5. Caenorhabditis elegans Strains

The wild type N2 Bristol strain of *C. elegans* and the *slo-1* knockout strain NM1968 *slo-1 (js379) V* were obtained from the *Caenorhabditis* Genetics Center, University of Minnesota. Worm stocks were maintained on nematode growth medium (NGM) plates pre-seeded with *E. coli* OP50 (*Caenorhabditis* Genetics Center) as a food source at 20 °C [22].

#### 4.5.1. Preparation of Assay Plates

Stock solutions were prepared in DMSO. Proper dilutions to achieve final concentrations were conducted in NGM agar after autoclaving at ~50 °C. Final DMSO concentration was 28 mM. This DMSO concentration does not have any effects on *C. elegans* locomotion [22,23]. All plates were seeded with ~100 μL *E. coli* OP50.

#### 4.5.2. Locomotion Assay: Number of Reversals Count

The setup for the reversal assay was as follows: 30–40 synchronized L1 stage larvae of N2 and NM1968 *slo-1 (js379) V* strains were transferred to NGM plates seeded with *E. coli* OP50 containing 50 μM of DHA or AST, 2 μM of PA, co-treatments with DHA or AST (50 μM) and PA (2 μM), and DMSO (vehicle control). The plates incubated for 42 h at 20 °C. At the end of the incubation period, L4 larvae were individually transferred to a plain un-seeded NGM plate and allowed to crawl for one min to acclimatize and allow for the removal of any residual bacteria that may be adherent to the worm. The reversals were then counted for 3 min. Statistical variations of reversal numbers in different treatments were compared using one-way-ANOVA.

### 4.6. Sprague Dawley Rats

Sprague Dawley rats (100–120 g) were purchased from Animal House Colony (Giza, Egypt) maintained on a standard laboratory diet and water ad libitum at the Animal House Laboratory, National Research Centre, Cairo, Egypt. All animals received humane care in compliance with the guidelines of the Animal Care and Use Committee of the National Research Centre, Cairo, Egypt.

To prepare PA-contaminated rat food, one kilogram of barley containing 465 mg PA was evenly and homogenously mixed with nine kilograms of the standard rat diet so that the final concentration of PA in this food mixture was 46.5 mg/kg.

Experimental rats were divided into eight groups (10 rats/group) as follows:

Group 1: Control saline (10 mL/kg, oral). Group 2: AST (12 mg/kg, oral) [46]. Group 3: DHA (250 mg/kg, oral). Group 4: AST (12 mg/kg, oral) + DHA (250 mg/kg, oral). Group 5: PA (46.5 mg/kg on diet). Group 6: PA (46.5 mg/kg on diet) + AST (12 mg/kg, oral). Group 7: PA (46.5 mg/kg on diet) + DHA (250 mg/kg, oral). Group 8: PA (46.5mg/kg on diet) + AST (12 mg/kg, oral) + DHA (250 mg/kg, oral). Groups received the above-mentioned treatments daily for four weeks. Animals were observed daily. After collection of blood sample, animals were euthanized by cervical dislocation, and samples of brain were dissected and homogenized for determination of neurotransmitters, TAC, MDA, and NO. Another portion of the brain of each animal was dissected and fixed in natural formalin 10% for histopathological studies.

#### 4.6.1. Histopathological Examination

Tissue specimens of rat brains of all experimental treatments were collected at the study end and fixed in neutral buffered formalin, processed by conventional method, embedded in paraffin, sectioned at 4–5 μm, and stained by haematoxylin and eosin (H & E, [47]).

#### 4.6.2. Determination of Brain Amino Acids and Monoamines

Each brain tissue was weighed and homogenized in 75% aqueous HPLC grade methanol (10% *v*/*v*). The homogenate was spun at 4000 rpm for 10 min, and the supernatant was divided into two halves; the first was dried under vacuum and used for GABA (γ-amino-butyric acid) and glutamate determination, whereas the second half was used for monoamine determination. Brain aspartate, glutamate, and GABA were detected by quantitative HPLC using the precolumn phenyl isothiocyanate (PITC, Edman’s Reagent) derivatization technique according to Heinrikson and Meredith’s 1984 method [48]. Brain monoamines were immediately extracted from the trace elements and lipids by the use of solid phase extraction CHROMABOND column NH2 phase cat. No. 730031. The sample was then injected directly into an AQUA column 150 mm × 4.6 mm, 5 μm C18, purchased from Phenomenex, USA under the following conditions: mobile phase 20 mM potassium phosphate, pH 2.5, flow rate 1.5 mL/min, UV 210 nm. Norepinephrine, dopamine, and serotonin were separated after 12 min, according to the method of Pagel et al. (2000) [49].

Calculations:

$$\text{Concentration}\;\mathrm{of}\;\text{monoamine}\;\mathrm{of}\;\text{standard}\;\left(\mu g\right)\;=\;\frac{\text{Area}\;\mathrm{of}\;\text{under}\;\text{curve}\;\times \;100}{\text{Area}\;\mathrm{of}\;\text{standard}\;\text{under}\;\text{curve}}$$

#### 4.6.3. Measurement of Total Antioxidant Capacity in Serum and Brain Homogenate

Serum and tissue TAC were determined according to the method described by Koracevic et al. (2001) [50].

#### 4.6.4. Determination of Nitric Oxide in Serum and Brain Homogenate

Serum and tissue NO were determined according to the method described by Montgomery and Dymock (1961) [51].

#### 4.6.5. Assessment of Lipid Peroxidation by Measurement of MDA in Brain Tissue

Brain tissue samples (0.05–0.1 g) were homogenized in phosphate buffer (pH 7.4) to give 20% (*w*/*v)* homogenate. This homogenate was centrifuged at 1700 rpm at 4 °C for 10 min. The supernatant was stored at −70 °C until analysis. This supernatant (20%) was used for the determination of lipid peroxidation according to the method described by Ruiz-Larrea et al. (1994) [52]. In brief, 4.5 mL working reagent: 1 volume of (0.8 g thiobarbituric acid dissolved in 100 mL of 10% perchloric acid) and 3 volumes of (20% trichloroacetic acid) were added to 0.5 mL of sample and incubated for 20 min in a boiling water bath then left to cool at room temperature before centrifugation at 3000 rpm for 5 min at 0 °C. The pink color was colorimetrically measured at wavelength 532 nm, against blank solution, which was prepared by the addition of 0.5 mL of distilled water to 4.5 mL working reagent, using a V-530 UV/Vis spectrophotometer (American German laboratory spectrophotometer: 1002, wave length range: 325–1000 nm, band width 2 nm, power: 220 V–50/60 Hz; 84 southaven Ave Medford NY 11763-3740 USA). The level of lipid peroxidation was expressed as nM MDA/g brain tissue.

#### 4.6.6. Statistical Analysis

The biochemical parameters were analyzed by repeated measures, one-Way ANOVA followed by Tukey’s test. Differences with *p* < 0.05 were considered statistically significant.

## 5. Conclusions

DHA and AST treatments effectively counteracted the PA-induced toxic effects and normalized most biochemical parameters in cell culture, in vitro, as well as in the nematode *C. elegans* and rat in vivo models. The in vivo counteracting effects of AST and DHA for PA’s BK channel antagonism might be attributed to restoring the normal levels of targeted neurotransmitters.

## Supplementary Materials

The following are available online at www.mdpi.com/1660-3397/14/11/208/s1, Figures S1–S8: Additional histopathology sections showing the PA-induced toxicity and DHA and AST protective effects in rat brains.

## Abbreviations

The following abbreviations were used in this manuscript:

AST	Astaxanthin
AUC	Area Under the Curve
BBB	Blood Brain Barrier
*C. elegans*	*Caenorhbdities elegans*
CNS	Central Nervous System
DA	Dopamine
DHA	Docosahexaenoic Acid
DMEM	Dulbecco’s Modified Eagle’s Medium
DMSO	Dimethyl Sulfoxide
ASP	Aspartate
GLU	Glutamate
GABA	Gamma-Amino Butyric Acid
5-HT	5-Hydroxytryptamine (Serotonin)
HPLC	High Performance Liquid Chromatography
iNOS	Inducible Nitric Oxide Synthase
NGM	Nematode Growth Medium
NE	Norepinephrine
NO	Nitric Oxide
MDA	Malondialdehyde
*P. crustosum*	*Penicillium crustosum*
PA	Penitrem A
TAC	Total Antioxidant Capacity
HPA	Hypothalamic–Pituitary–Adrenal Axis
PITC	Phenyl isothiocyanate
