# Supplementary material for: Astaxanthin and Docosahexaenoic Acid Reverse the Toxicity of the Maxi-K (BK) Channel Antagonist Mycotoxin Penitrem A
Source: Mar Drugs. 2016 Nov 9;14(11):208. doi: 10.3390/md14110208 (PMC5128751; doi:10.3390/md14110208)
Supplement: Supplementary file 1 [file marinedrugs-14-00208-s001.docx]

Supplementary Materials: Astaxanthin and Docosahexaenoic Acid Reverse the Toxicity of the Maxi-K (BK) Channel Antagonist Mycotoxin Penitrem A

Amira A. Goda, Khayria M. Naguib, Magdy M. Mohamed, Hassan A. Amra, Somaia A. Nada, Abdel- Rahman B. Abdel-Ghaffar, Chris R. Gissendanner and Khalid A. El Sayed


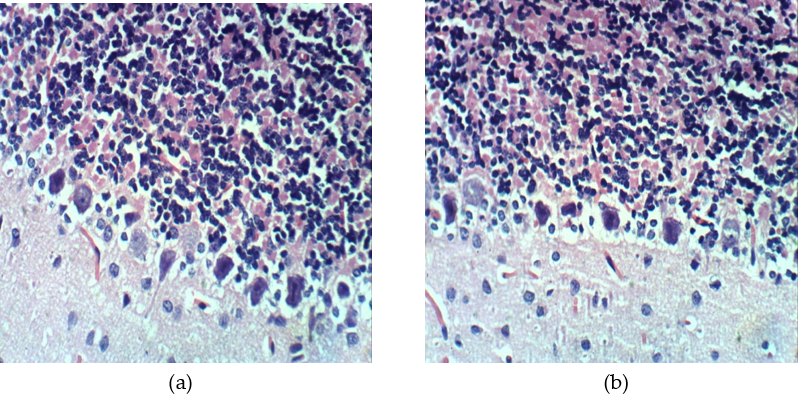


**Figure S1.** Histology of brain sections for vehicle-treated control group rats: (**a**) Cerebellum section showing no histopathological changes with normal Purkinje cells (H & EX400); (**b**) Cerebellum section showing no histopathological changes with normal Purkinje cells (H & EX400).


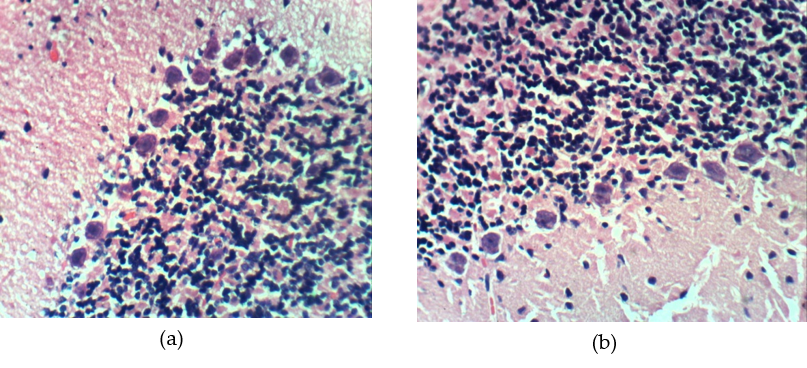


**Figure S2.** Histopathology of astaxanthin (AST)-treated rat brain sections. (**a**) AST-treated rat cerebellum section showing normal Purkinje cells (H & EX400); (**b**) AST-treated rat cerebellum showing no histopathological changes with normal Purkinje cells (H & EX400).

**
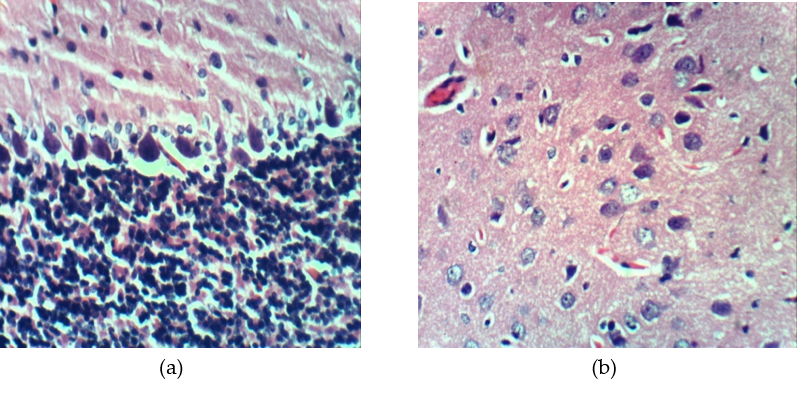
**

**Figure S3.** Histopathology of docosahexaenoic acid (DHA)-treated rat brain sections. (**a**) Cerebellum section showing no histopathological changes with normal Purkinje cells (H&EX400); (**b**) Cerebrum section showing no histopathological changes (H&EX400).

**
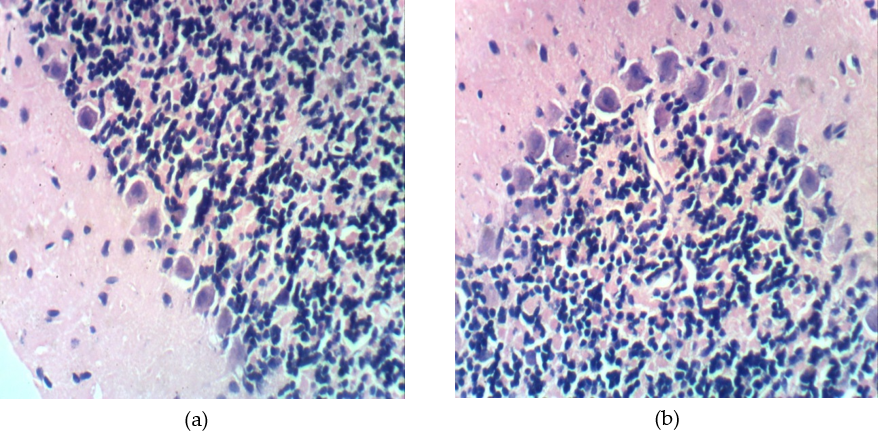
**

**Figure S4.** Histopathology of combined DHA and AST-treated rat brain sections. (**a**) DHA/AST-treated cerebellum section showing no histopathological changes with normal Purkinje cells (H & EX400); (**b**) Cerebellum section showing no histopathological changes with normal Purkinje cells (H & EX400).


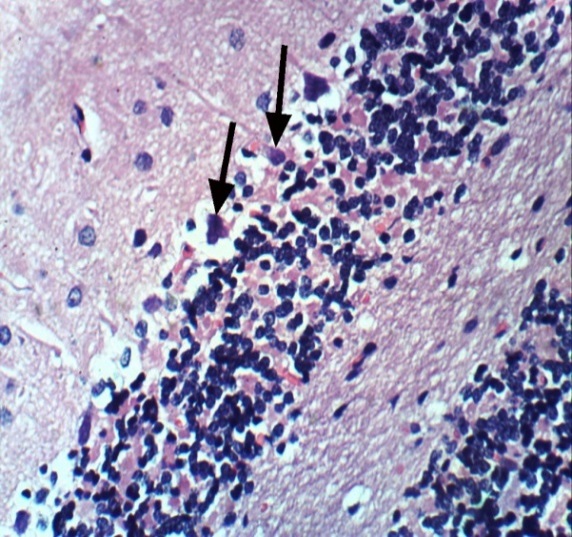


**Figure S5.** Histopathology of penitrem A (PA)-treated rat cerebellum sections. The arrows show necrosis of Purkinje cells (H & EX400).


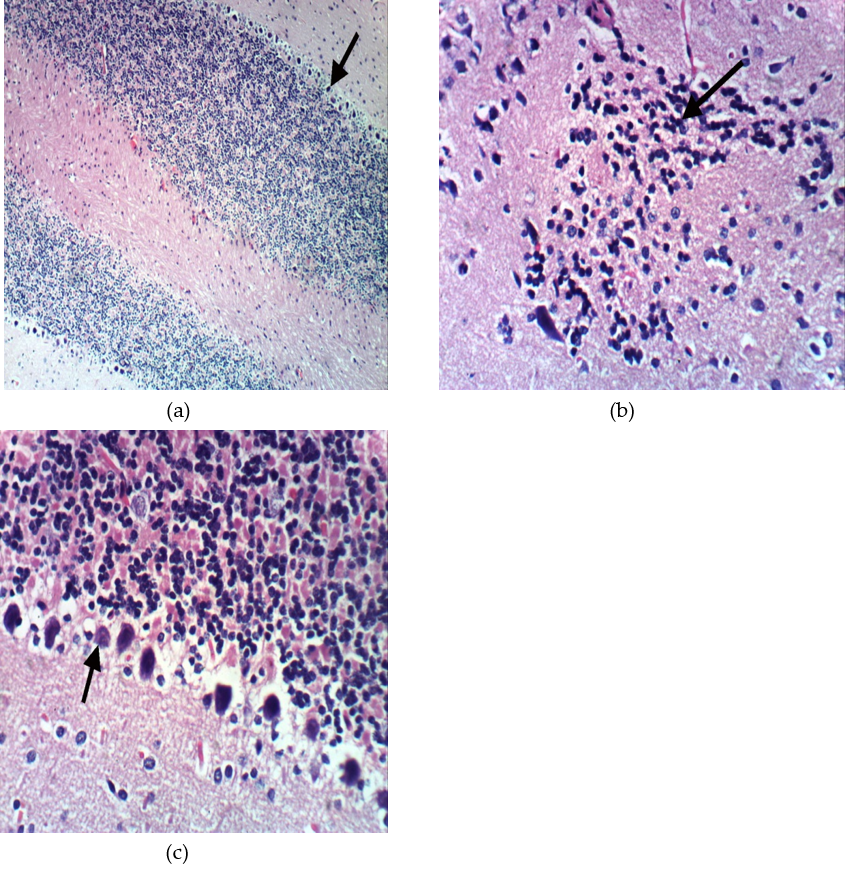


**Figure S6.** Histopathology of combined AST and PA-treated rat brain sections. (**a**) AST and PA-treated rat cerebellum section, the arrows showing necrosis of Purkinje cells (H & EX100); (**b**) Cerebrum section, the arrows showing focal gliosis (H&EX400). (**c**) Cerebellum section with arrows showing necrosis of some Purkinje cells (H&EX400).


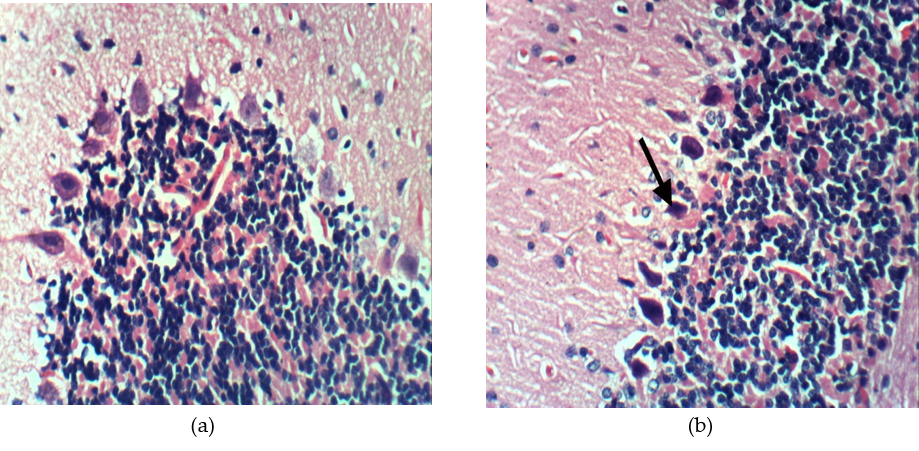


**Figure S7.** Histopathology of combined DHA and PA-treated rat brain sections. (**a**) DHA and PA-treated rat cerebellum showing normal Purkinje cells (H & EX400); (**b**) Cerebellum section with arrows showing necrosis of some Purkinje cells (H & EX400).


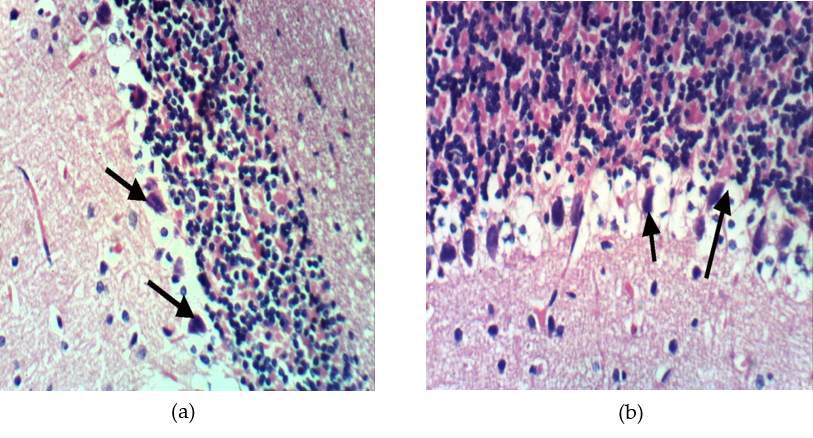


**Figure S8.** Histopathology of combined DHA, AST, and PA-treated rat brain sections. (**a**) DHA, AST, and PA-treated rat cerebellum section with arrows showing necrosis and decreased number of Purkinje cells (H & EX400); (**b**) Cerebellum section of rat with arrows showing necrosis of Purkinje cells and vacuolization of molecular cell layer (H&EX400).
